# Disrupted brain network dynamics and cognitive functions in methamphetamine use disorder: insights from EEG microstates

**DOI:** 10.1186/s12888-020-02743-5

**Published:** 2020-06-24

**Authors:** Tianzhen Chen, Hang Su, Na Zhong, Haoye Tan, Xiaotong Li, Yiran Meng, Chunmei Duan, Congbin Zhang, Juwang Bao, Ding Xu, Weidong Song, Jixue Zou, Tao Liu, Qingqing Zhan, Haifeng Jiang, Min Zhao

**Affiliations:** 1 Shanghai Mental Health Center, Shanghai Jiao Tong University School of Medicine, 600 Wan Ping Nan Road, Shanghai, 200030 China; 2 Yunnan Institute on Drug Dependence, Kunming, Yunnan China; 3 Institute of Higher Education, Beijing University of Technology, Beijing, China; 4 Shanghai Bureau of Drug Rehabilitation Administration, Shanghai, China; 5 Department of Health, Yunnan Bureau of Drug Rehabilitation Administration, Kunming, Yunnan China; 6 Yunnan Third Compulsory Drug Dependence Rehablitation Center Hospital, Kunming, Yunnan China; 7 Shanghai Key Laboratory of Psychotic Disorders, Shanghai, China; 8 Institute of Psychological and Behavioral Science, Shanghai Jiao Tong University, Shanghai, China; 9 CAS Center for Excellence in Brain Science and Intelligence Technology (CEBSIT), Chinese Academy of Sciences, Shanghai, China

**Keywords:** Methamphetamine use disorder, EEG microstate, Brain network dynamics, sLORETA, Drug use history, Attention bias

## Abstract

**Background:**

Dysfunction in brain network dynamics has been found to correlate with many psychiatric disorders. However, there is limited research regarding resting electroencephalogram (EEG) brain network and its association with cognitive process for patients with methamphetamine use disorder (MUD). This study aimed at using EEG microstate analysis to determine whether brain network dynamics in patients with MUD differ from those of healthy controls (HC).

**Methods:**

A total of 55 MUD patients and 27 matched healthy controls were included for analysis. The resting brain activity was recorded by 64-channel electroencephalography. EEG microstate parameters and intracerebral current sources of each EEG microstate were compared between the two groups. Generalized linear regression model was used to explore the correlation between significant microstates with drug history and cognitive functions.

**Results:**

MUD patients showed lower mean durations of the microstate classes A and B, and a higher global explained variance of the microstate class C. Besides, MUD patients presented with different current density power in microstates A, B, and C relative to the HC. The generalized linear model showed that MA use frequency is negatively correlated with the MMD of class A. Further, the generalized linear model showed that MA use frequency, scores of Two-back task, and the error rate of MA word are correlated with the MMD and GEV of class B, respectively.

**Conclusions:**

Intracranial current source densities of resting EEG microstates are disrupted in MUD patients, hence causing temporal changes in microstate topographies, which are correlated with attention bias and history of drug use.

## Background

Drug dependence is a severe medical and public health challenge globally. Methamphetamine (MA), one of the amphetamine-type stimulants, induces toxic effects on the brain, such as oxidative stress, mitochondrial toxicity, excitotoxicity and neuroinflammation [1]. Overall, these effects disrupt the structure and functioning of the brain. Previous studies reported that abuse of MA is associated with extensive cognitive impairment, encompassing the executive function, working memory, problem-solving, and attention deficit [2, 3]. It has been shown that these impairments are correlated to brain imaging changes in MA-dependent patients [2, 4]. Some of the brain regions damaged by MA include the dorsal prefrontal cortex, medial prefrontal cortex, anterior cingulate cortex, striatum, and insula. Notably, magnetic resonance imaging (MRI) can effectively diagnose and evaluate cognitive impairment in MA dependent patients. Besides, the typical characteristics of drug use history are also closely associated with cognitive impairment and structural changes of the brain of MA dependent patients [5–7].

Information processing by the brain is associated with the temporal and spatial dynamics of the whole brain resting network [8, 9]. Coordination and integration of multiple brain regions is an essential basis of understanding cognitive function [10, 11]. Studies have indicated that the spatial-temporal characteristics of brain networks are beneficial in defining and understanding the elements of cognitive impairment [12–14]. However, most previous studies on cognitive function in MA-dependent patients have focused on the behavioural changes and alterations in the spatial structure of the brain. Meanwhile, only a few have explored the temporal characteristics of cognitive processing [7, 15, 16]. Therefore, there is a dearth of information on cognitive impairment in patients with substance use disorder, such as methamphetamine use disorder (MUD).

In recent years, EEG microstate methods are increasingly being used to study the spatial and temporal resting state dynamics of brain networks [17]. Resting state EEG displays as periods of potential topographies and shows quasi-stable configurations called functional microstates [18]. Functionally, EEG microstates represent the momentary local states and may indicate spontaneous fluctuations in activity in large scale brain networks [19]. Also, EEG microstates reflect basic brain activity in spontaneous and event-related EEG researches [20, 21]. Most studies have clustered EEG microstate topographies into four standard microstate maps, and these categories represent significant brain configuration networks [17]. The various microstates are associated with different types of cognitive processes [22–26], suggesting that microstates might effectively analyze cognitive deficits resulting from a mental disorder and addiction. Britz and colleagues used simultaneous EEG-fMRI techniques to explore the correlation between microstates and distinct fMRI resting-state networks [22]. Previous research showed that the four microstates marked A, B, C, and D correspond to resting-state networks that had previously been identified to be associated with phonological processing, the optical network, the saliency network, and attention, respectively [22]. Besides, these four microstate classes are correlated with the various aspects of the default mode network [27], which play an important cognitive role in psychiatric diseases [28]. Notably, studies focusing on the association between resting-state EEG and cognition impairment in MUD patients mainly use the frequency and entropy analysis methods. The results of these studies suggested an important relationship between EEG signals and cognitive function [29–32]. The micro-state analysis method is beneficial in extracting the temporal information of EEG signals.

The findings of previous studies on several psychiatric and neurological disorders suggest that aberrant EEG microstates reflect abnormal information processing capabilities during the cognitive process [17, 21, 33–35]. However, the temporal dynamics of the brain networks in drug users have not been analyzed using the resting EEG microstate method. The present study, therefore, used this method to study the association between disrupted brain dynamic networks and cognitive function of MA users and the potential effect of drug use history. Through this, it is possible to provide novel strategies for understanding the dynamics of cognitive function disruption in MA-dependent patients and other drug users.

Therefore, this study aimed at investigating the resting state EEG microstates in MA-dependent patients. Since structural alterations have been identified using other brain imaging methods (i.e. MRI and PET), we hypothesized that relative to controls, a disrupted resting state EEG microstate may develop in MA-dependent patients. Besides, these disruptions could be associated with cognitive impairment and the characteristics of drug use history.

## Methods

### Participants

This study is a part of a larger project investigating the objective marker system for diagnosis of MUD and relapse warning among MA users in China. We recruited 57 participants with MUD from two compulsory rehabilitation centers in Shanghai and Yunnan provinces, China. All patients in the rehabilitation centers from June 2017 to August 2018 were invited to participate in the study. The recruitment criteria included: (1) minimum age of 18 years; (2) met the requirements of the fifth edition of the Diagnostic and Statistical Manual of Mental Disorders (DSM-5) for MUD; (3) used MA in the three months before recruitment in the study (4) normal vision and hearing, or within the normal range after correction (5) right-handed. The exclusion criteria were (1) diagnosis of other substance use disorders or other axes I psychiatric disorders; (2) history of head injury, neurological disorders, or loss of consciousness; (3) patients on any antidepressant or/and antipsychotic medication.

An additional 30 healthy controls (HC) were recruited from the local community through advertisement and word-of-mouth. The recruitment was conducted between June 2017 and November 2018, after which HC were matched with the MA groups by gender and age. Regarding the choice of matching variables, previous studies have shown that age significantly affects resting EEG signals and cognitive function [36]. Besides, previous studies have suggested that EEG signals and cognitive functions differ between males and females [37], so both age and gender were used as matching variables in this study. The enrollment of HC was done simultaneously with that of patients, so the matching process was part of recruitment. For HC, the exclusion criteria included (1) current substance abuse or dependence; (2) history of substance dependence other than nicotine; (3) a current or previous mental illness (or/and treatment); (4) history of head injury, neurological disorders, or loss of consciousness; (5) without normal vision or hearing, or out of range after correction; (6) left-handed. The study protocol was implemented in compliance with the principles of the Declaration of Helsinki and approved by the Ethical Committee of the Shanghai Mental Health Center. Informed consent was signed by all subjects before participation in this research.

### Measures

#### Socio-demographic variables

After obtaining informed consent, trained research assistants at the Shanghai Mental Health Center used a Chinese version of the Addiction Severity Index (ASI-C) to collect baseline information from the subjects. The information was collected via face to face interviews in a private environment to avoid disturbance from others. Patients in the baseline study were interviewed after detoxification. The variables used included basic demographic characteristics (age, gender), history of MA use (accumulated years of MA use, frequency, and quantities of daily use), and alcohol use history. The psychometric properties of ASI-C have been validated in previous studies [38].

#### CogState battery

CogState Battery is a computerized cognitive tool designed to assess cognitive function, and consists of Two Back Task (TWOB, working memory), International Shopping List Task (ISL, verbal learning and memory), the Groton Maze Learning Task (GML, problem solving/ error monitoring), Social Emotional Cognition Task (SEC, social cognition), and Continuous Paired Association Learning Task (CPAL, spatial working memory). Detailed procedures and rules of the above paradigms have been provided in past research [39]. In these five tasks, the ISL score is the total correct response, the TWOB and SEC scores are the rates of accurate answers, while the GML and CPAL sores are the total error numbers. Good reliability and validity of the CogState Battery (Chinese version) were verified previously [39].

#### Stroop task

Attention bias was assessed by the MA Addiction Stroop Task [40]. Two target words ((“ICE” means “MA” and “SKATING” is the nickname for how Chinese drug users refer to taking MA)) and two neutral words ((“MESSY GRASS” and “RESOLVE”)) were involved in this paradigm.

The subjects were asked to press the buttons according to the color of the words presented (red, green, yellow, and blue), and ignore the meaning of the words. Each of the four words was presented eight times and was shown on the screen for 3000 ms each time. The word appeared randomly during the test, and the same word was set not to appear three times consecutively. This task included 64 MA-related word trials and 64 neutral word trials. Two outcomes were measured for this task: (1) Reaction time, which was obtained from button presses of the dominant hand on four color-marked buttons; (2) Errors, calculated as a ratio of the number of wrong color pressed by the subjects to the total number of trials recorded. More detailed information about this paradigm can be found in previous articles published by colleagues in our laboratory [40]. All assessments were done following the standardized instructions handbook by a trained researcher. The fixation cross and words were displayed on black background 75 cm from the eyes.

#### EEG recording and processing

The scalp EEG data were recorded from a high-density 64-channel sintered Ag/AgCl electrode cap (BrainCap; Asiacut, Germany). Two vertical electrooculography (EOG) electrodes were positioned above and below the right eye, and the other two horizontal EOG electrodes were placed on the outer canthi of both eyes. The reference electrode was located at the tip of the nose, while the ground electrode was placed on the forehead. The EEG was sampled continuously at a sampling frequency of 1000 Hz, and all impedances were kept below 5 kΩ. EEG was recorded in the eyes-closed resting state for 5–6 min.

EEG data were bandpass filtered at 0.1–70 Hz and eye movement artifacts were removed using independent component analysis. Manual screening for other artifacts was then conducted. In accordance with the steps detailed in previous research [18, 19], data were subsequently segmented into artifact-free 2 s epochs before microstate analysis, digitally band passed at 2–20 Hz and down-sampled to 250 Hz. The first 20 artifact-free epochs of each study participant were after re-referenced to the average reference and used for the analysis of resting EEG microstates. EEG data were processed using EEGLAB 13.0b [41] in MATLAB2013b (The MathWorks, Inc., Natick, US).

### Microstate analysis

EEG epochs were transformed into global scalp sequences of momentary potential map distributions to analyze EEG microstates. We used the standard strategies for analysis of microstates described in previous researches [42–44]. The fitted microstate class topographies were computed using a modified version of the k-means spatial cluster analysis based on previous studies [19, 45]. Original topographies were clustered into four microstate classes according to the findings of a large normative study in healthy populations [19]. We then performed a two-steps k-means clustering analysis. At the individual level (first-step), global field power peaks of each participant were submitted for k-means clustering analysis, and four microstate classes were obtained per individual, which is consistent with previous studies [46–48]. In the second step, also known as the group-level, the dominant topographies across all individuals and groups were clustered to obtain four mean microstate classes for each group. Fitting of microstates was then conducted to calculate the related parameters, including mean microstate duration (MMD), the ratio of total time covered (RTT), global explained variance (GEV), and occurrence of each microstate in each participant. The MMD indicates the mean times of each microstate class, RTT indicates the mean ratio of total time covered by each of the four microstates, while GEV quantifies the extent to which each of the four microstates describes the data. Microstate analysis was performed using the free academic software Cartool (https://sites.google.com/ site/cartoolcommunity/) [49].

### Statistical analysis

The Student’s T-test or the Chi-square test was used to test for differences in baseline characteristics between patients with MUD and HC. Data with non-homogenous variance and/ or not normally distributed were analyzed using non-parametric tests. Two-way Analysis of Variance, performing with microstate class as within-subject factors and the subject group as between factor, was used to compare each microstate-related measurement. Two-tailed Student’s T-test was then used to compare significant results from each group. All microstate related parameters of each microstate were tested four times. The corrected T-test alpha value was set to 0.0125 based on the Bonferroni correction method, and the generalized linear regression model was used to explore the correlation between significant microstate related parameters and other factors (i.e. drug history, CogState Battery and Addiction Stroop Task).

We then investigated the intracerebral current sources of each microstate classes which best differentiated the groups in microstate-related parameters using standardized low-resolution brain electromagnetic tomography (sLORETA) [50]. The intracerebral current sources were calculated from the individual microstate templates of each class for all MA-dependent patients and HC. Electrode coordinates were transferred to 6239 cubic voxels corresponding to cortical grey matter and hippocampi in the sLORETA inverse solution space. Differences between the two groups were assessed by voxel-by-voxel unpaired sample log of F-ratio-tests for each microstate class. The statistical non-parametric mapping randomization test was used to correct critical probability threshold values for multiple comparisons. A total of 5000 permutations were used to determine the significance of each randomization test. Source analysis was performed using the sLORETA software (http://www.uzh.ch/keyinst/loreta.htm).

## Results

### Description of member characteristics

Five individuals (including 2 MA-dependent users and 3 HC) who had more than five bad channels were excluded in the subsequent analysis. To assess the sample size, we performed a power analysis using the microstates MMD difference between the two groups. The analysis results revealed that our sample size has a power of 0.82, suggesting reliability of the subsequent results. Table 1 summarizes the demographic characteristics and performance of each cognitive task (Cogstate Battery and Stroop Task) for both MA and HC samples. There were no significant differences in gender (t = 0.00, *p* = 0.96) and mean age (t = 0.53, *p* = 0.59) between MA-dependent patients and HC.

**Table 1:** Baseline characteristics of participants

	MA patients (*n* = 55)	HC (*n* = 27)	t value/χ^2^	df	p
Male gender	67.3%	66.7%	0.00		0.96
Mean age (SD)	29.56 ± 4.90	30.24 ± 6.55	−0.53	80	0.59
Education years	8.81 ± 2.43	9.48 ± 2.34	−1.19	80	0.24
Drug use history					
Alcohol co-use^a^	52.7%	52.4%	0.01		0.94
Accumulated years of MA use (SD)	5.00 ± 3.47	–			
MA use frequency					
Every day	32.7%	–			
3–5 days per week	56.4%	–			
1 day per week	10.9%	–			
1 day per month	0	–			
MA use disorderc					
mild	0	–			
moderate	16.4%	–			
severe	83.6%	–			
MA use dose (SD), gram	0.68 ± 0.48	–			
Stroop task					
Reaction time of MA word	755.86 ± 79.62	738.22 ± 113.17	0.82	80	0.42
Reaction time of neutral word	747.76 ± 80.44	740.73 ± 113.75	0.32	80	0.75
Error rate of MA word	0.08 ± 0.08	0.03 ± 0.02	3.19	80	0.00**
Error rate of neutral word	0.07 ± 0.08	0.03 ± 0.03	2.51	80	0.01*
Cogstate Battery					
TWOB (accuracy rate)	1.01 ± 0.22	1.03 ± 0.22	−0.38	80	0.70
GML (total error)	60.84 ± 19.10	47.81 ± 28.01	2.48	80	0.02*
ISL (total correct)	20.56 ± 5.23	23.10 ± 5.87	−2.00	80	0.05
SEC (accuracy rate)	0.92 ± 0.21	0.98 ± 0.24	−1.16	80	0.25
CPAL (total error)	92.08 ± 51.10	52.86 ± 49.84	3.29	80	0.00**

^a^Use alcohol in the past 30 days before recruited

^b^Use tobacco in the past 30 days before recruited

^c^Severity was diagnosed through DSM-5

Abbreviations: MA, methamphetamine; HC, healthy controls; SD, standard deviation

^*^p<0.05; ^**^p<0.01

### Resting state EEG microstates

**Fig. 1:**
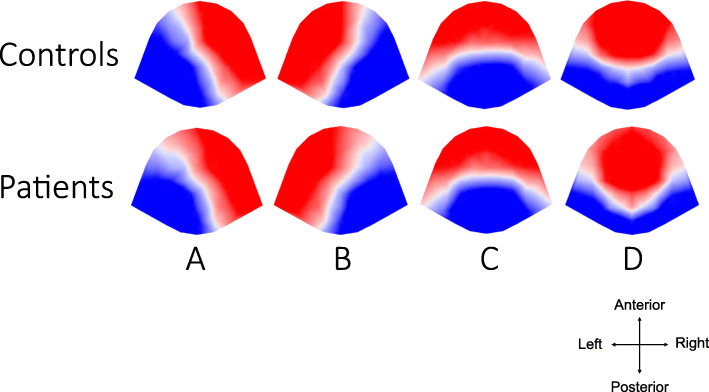
Topographical microstate maps. The figure shows spatial configuration of the four microstate classes (**a**–**d**). Upper row: microstate configurations of healthy controls. Lower rows: microstate configurations of patients with methamphetamine use disorder. Different colors represent the different polarities. The maps are presented as seen from above angle

The topography classes observed in this study were similar to four classes reported in the previous literatures, and were labeled microstate A, B, C, and D accordingly (Fig. 1) [18, 19, 51]. The four microstate topographies included one with a right anterior-left posterior orientation (microstate A), one with a left anterior- right posterior orientation (microstate B), one with a central anterior-posterior orientation (microstate C), and one with a fronto-central extreme location (microstate D).

The microstate parameters of the four microstate classes are summarized in Table 2. No significant group effects were identified in the four microstate parameters, but a significant interaction between the groups and microstate classes were observed for GEV (F = 3.99, *p* = 0.01), MMD (F = 2.67, *p* = 0.05), and RTT (F = 3.31, *p* = 0.02). Subsequent analysis indicated significantly lower MMD (t = − 2.77, corrected *p* = 0.03) in microstate class A of MA patients relative to HC. For microstate B, we found a significantly lower MMD (t = − 3.30, corrected p = 0.01) and GEV (t = − 3.19, corrected p = 0.01) in MA patients relative to HC. In contrast, microstate class C presented with significantly higher GEV (t = 2.61, corrected *p* = 0.04) in MA patients compared to HC.

**Table 2:** Microstate parameter results summary

	MA patients		HC		t-value	df	*p*-value	Corrected p-value
	Mean	SD	Mean	SD				
Map A								
MMD	37.42	2.56	39.54	4.37	−2.77	80	0.01**	0.03*
RTT	0.18	0.05	0.21	0.07	−2.23	80	0.03	0.11
GEV	0.11	0.05	0.13	0.07	−1.49	80	0.14	
Occurrences	4.79	0.98	5.08	1.27	−1.14	80	0.26	
Map B								
MMD	37.97	2.91	40.29	3.15	−3.30	80	0.00**	0.01**
RTT	0.19	0.05	0.21	0.04	−1.81	80	0.07	
GEV	0.11	0.04	0.14	0.04	−3.19	80	0.00**	0.01**
Occurrences	4.91	0.92	5.29	0.71	−1.89	80	0.06	
Map C								
MMD	46.83	5.53	45.05	2.99	1.56	80	0.12	
RTT	0.30	0.07	0.27	0.05	1.99	80	0.05	0.20
GEV	0.26	0.09	0.21	0.06	2.61	80	0.01*	0.04*
Occurrences	6.29	1.00	5.88	0.85	1.83	80	0.07	
Map D								
MMD	47.16	8.14	46.10	8.58	0.54	80	0.59	
RTT	0.29	0.09	0.26	0.10	1.36	80	0.18	
GEV	0.23	0.10	0.21	0.12	0.80	80	0.43	
Occurrences	5.92	1.22	5.50	1.25	1.45	80	0.15	

### EEG microstate source differences

Analysis of current density power by sLORETA showed significant differences in microstates A, B, and C between MA patients and HC. For microstate A, suprathreshold cortical voxels (Threshold: Log-F = 0.70, *p* = 0.05) displayed increased activation of current density power in the inferior parietal lobule, precentral gyrus, middle frontal gyrus, middle temporal gyrus, superior temporal gyrus, superior parietal lobule, supramarginal gyrus, and insula (Table S1 and Fig. S1 in the Supplement). For microstate B, suprathreshold cortical voxels (Threshold: Log-F = 0.76, *p* = 0.05) displayed increased activation in the regions of the inferior parietal lobule, middle occipital gyrus, inferior frontal gyrus, cuneus, precuneus, superior occipital gyrus, supramarginal gyrus, and angular gyrus among others (Table S2 and Fig. S2 in the Supplement). For microstate C, increased activations (Threshold: Log-F = 0.80, p = 0.05) were observed within the inferior frontal gyrus, inferior temporal gyrus, cingulate gyrus, posterior cingulate, and the parahippocampal gyrus among others, as shown in Table S3 and Fig. S3.

### Relationships between microstate parameters and other factors

A generalized linear model of class A indicated a negative correlation between MA use frequency (β = − 0.18, 95%CI = -0.74 to − 0.06, *p* = 0.02) and MMD. In Class B, a generalized linear model also indicated that MA use frequency (β = − 0.57, 95%CI = -0.96 to − 0.19, *p* = 0.00), scores of Two-back task (β = 0.45, 95%CI = 0.12 to 0.77, *p* = 0.01), an error rate of MA word (β = − 5.35, 95%CI = -8.52 to − 2.17, p = 0.00) were correlated with MMD. In class B also, we found that the MA use frequency (β = − 0.11, 95%CI = -0.17 to − 0.06, p = 0.00), scores of Two-back task (β = 0.06, 95%CI = 0.01 to 0.11, p = 0.01), and the error rate of MA word (β = − 0.58, 95%CI = -1.05 to − 0.10, *p* = 0.02) were correlated with GEV. None of other variables, including drug history, CogState Battery, and Addiction Stroop Task were found correlated with the MMD of class A, MMD of class B, or GEV of class B.

Also, no correlation was observed between the GEV of class C and drug history, CogState Battery, and Addiction Stroop Task.

## Discussion

To the best of our knowledge, this is the first study to investigate the resting state EEG microstate abnormality in drug abuse patients. Also, the present study is the first to explore the correlation between the alterations in brain resting network and cognitive functions (i.e. CogState Battery and Addiction Stroop Task), along with drug use history (i.e. accumulated years of MA use, frequency of MA use, daily dosage, and severity of MA use disorder). Here, we found that the temporal microstate profiles of MA-dependent patients are different from those of HC, and these microstate deviations could be explained by changes in intracranial current source densities. Moreover, our findings suggest that MA use frequency is associated with changes in microstates A and B, while variations in Addiction Stroop Task are correlated with changes in microstate B.

### Microstate

Microstates reflect the dynamic synchronization of neuronal functional networks. Relative to HC, we found that MA-dependent patients have altered microstates A, B, and C, but microstate D is comparable between the two groups. Although results on brain network studies of MA users are difficult to compare directly due to the different techniques and analytical methods adopted, the findings of several previous studies are consistent with our results. As Ahmadlou and his colleagues reported, chronic MA abusers exhibited disrupted Small-World brain network and global network deficit at the gamma band [52]. Functional Magnetic Resonance Imaging also revealed abnormal functional connectivity, which correlates with psychotic symptoms of MA patients [53]. Therefore, our results on brain networks partially concur with those of previous studies.

The various microstate parameters provide information about the underlying neural generators [21]. For instance, MMD is indicative of the stability of a microstate and its underlying neural assemblies [54, 55]. Relative to HC, we found that the MMD of MA-dependent patients is significantly shorter for microstates A and B. In contrast, microstates C and D were not significantly different between the two groups. This result suggests that MA-dependent patients have underlying instability in microstates A and B, but are more stable in microstates C and D. Similarly, patients with schizophrenia have reduced MMD in microstates A and B [56, 57], suggesting an electrophysiological similarity in the mechanism of schizophrenia and MA-induced psychosis [58–60]. GEV reflects the time covered in each microstate class and its underlying neural generators [21]. Relative to HC, we found that the GEV of patients with MA use disorder exhibited higher in microstate C, but lower for microstate B. Notably, patients with obsessive-compulsive disorder also have abnormalities in these two microstate classes [61]; that is, the occurrence of these microstates was significantly different from those of HC. Previous studies have also suggested similarities in the manifestation of visual and salience network networks in patients with obsessive-compulsive disorder and addiction [62–67]. Therefore, abnormalities in these brain networks may partly explain the alteration in the parameters in microstates B and C in these two populations [22].

### Spatial configuration of microstate classes and the relationship between parameters

The findings of researches that combined resting state EEG and fMRI suggested that EEG microstate classes mirror various large-scale resting-state networks, and thus reflected the specific functional state of the mental process [22, 68]. For instance, Microstate A, which is closely associated with blood oxygen level-dependent (BOLD) activation of bilateral superior temporal and parietal cortex, was suggested to reflect the sensorimotor or auditory network [22, 69]. We conducted the sLORETA analysis to interpret the changes in microstate class A in MA-dependent patients and observed hyper-activation of the superior temporal and parietal area in these patients, relative to HC. Besides, we found that the microstate MMD of MA dependent patients was shorter than that of HC. Interestingly, we found that the MMD of microstate A was negatively correlated with the MA use frequency. Previous studies have reported that MA abuse impairs sensorimotor cortical plasticity and causes deficits in behavioural performance [70, 71]. The frequency of MA use was found to be associated with the volume of cortical gray matter [72]. Daumann and colleague suggested that lifetime use of MA is negatively correlated with individual gray matter [73]. Ruan and colleague also indicated that accumulated years of MA use are negatively correlated with the thickness of gray matter in the right superior temporal cortex [5]. These findings indicate that the aberrance of parameters of microstate A may reliably indicate the severity of MA use.

As one of the specific predictors of relapse, attention bias was assessed by the MA Addiction Stroop Task. We found that attention bias is correlated with aberrant temporal functioning of microstate B. The microstate B was found associated with negative BOLD activation in the striate and extrastriate cortex [22]. Similar to our findings, a previous study reported that the MMD of microstate B is significantly lower in patients with mild spastic diplegia [74]. Besides, patients with moderate spastic diplegia display various visual categorization impairments and attention deficits [75, 76]. Collectively, microstate class B may play an essential role in visual perception and attention. Therefore, the risk of attention bias in MA-dependent patients can be assessed by measurement of microstate B. Besides, the present study also demonstrated that MA use frequency is correlated with changes in the GEV and MMD of microstate B. Previous studies have shown that drug use history is associated with deficits in brain structure and cognitive impairment in MA-dependent patients [5, 16]. sLORETA analysis of microstate B further suggested that regions related to visual processing (including middle occipital gyrus, cuneus, superior occipital gyrus, and the precuneus) are hyperactive in patients with MA use disorder. Therefore, it is reasonable to conclude that the microstate B may be an explanation of cue-reactivity in MA-dependent patients. Interestingly, we also identified a positive correlation between working memory performance and the GEV of microstate B. It has been suggested that the parahippocampal gyrus and fusiform gyrus (specific brain area of visual network) transmit information relevant to the visual cue. Notably, their spatial activation patterns during selective maintenance of the cue-related picture type match those during the processing of visual working memory [77]. Therefore, abnormalities in microstate B also partially explain the severity of working memory deficits in patients with MA use disorder. Nevertheless, aberrant levels of microstate parameters do not indicate variations in the function of the corresponding brain region [57, 78]. Whether the behavioral performance is regulated by the balance between different EEG microstates and whether the performance is related to a single microstate parameter within a reasonable range are subjects of future research.

### Limitations and strengths

There are some limitations to this study. Although the study participants were recruited from two regions, our sample size was relatively small. Therefore, future studies should use a larger sample size to increase the statistical power and the robustness of the research, and hence generate more reliable results for clinical applications. Second, although we screened all the possible patients in the two rehabilitation centers, our study population may not represent the characteristics of the MA patients at all various treatment states and different abstinence periods. Another shortcoming is that we did not simultaneously record MRI data, which provides a more direct picture of the activities in the brain regions. Therefore, we could not determine the correlation between EEG microstates and the activity of distinct brain areas. In addition, due to the cross-sectional nature of our study, the conclusion that the aberrance observed in the microstates resulted from MA dependence should be made with more caution. As such, a longitudinal study should be done to verify our findings. The strengths of this study are as follows. Firstly, the gender and age of our patient group and the HC were sufficiently matched, and therefore no significant differences between the groups. Secondly, the low-cost and noninvasive EEG recording used to detect the high time resolution network dynamics of the brain is reliable; hence the results are applicable in clinical practice.

## Conclusions

Our study reveals that the microstate topographies were aberrant in patients with MUD. In general, the present study is the first to explain changes in the microstates of drug dependence patients and its correlation with relevant clinical features. Therefore, we provide preliminary evidence for exploring whether EEG microstates could be significant indicators of cognitive disruption in substance-dependent patients.

## Supplementary information

**Additional file 1: Table S1.** Brain regions that showed increased activation of microstate A at rest in patients with methamphetamine use disorder. **Table S2.** Brain regions that showed increased activation of microstate B at rest in patients with methamphetamine use disorder. **Table S3.** Brain regions that showed increased activation of microstate C at rest in patients with methamphetamine use disorder. **Fig. S1.** sLORETA differences in the cortical distribution of electrical activity sources of microstate class A between patients with methamphetamine use disorder and health controls. Significant differences are displayed (yellow = patients>controls). **Fig. S2.** sLORETA differences in the cortical distribution of electrical activity sources of microstate class B between patients with methamphetamine use disorder and health controls. Significant differences are displayed (yellow = patients>controls). **Fig. S3.** sLORETA differences in the cortical distribution of electrical activity sources of microstate class C between patients with methamphetamine use disorder and health controls. Significant differences are displayed (yellow = patients>controls).

## Data Availability

The datasets used and/ or analyzed during the current study are available from the corresponding author on reasonable request and with consent from the Institutional Review Board (IRB).

## Abbreviations

ASI-C: Chinese version of the Addiction Severity Index
BOLD: Blood oxygen level-dependent
CPAL: Continuous Paired Association Learning Task
DSM-5: The fifth edition of the Diagnostic and Statistical Manual of Mental Disorders
EEG: Electroencephalogram
EOG: Electrooculography
GEV: Global explained variance
GML: Groton Maze Learning Task
HC: Health controls
ISL: International Shopping List Task
MA: Methamphetamine
MMD: Mean microstate duration
MRI: Magnetic resonance imaging
MUD: Methamphetamine use disorder
PET: Positron emission tomography
RTT: Ratio of total time covered
SD: Standard deviation
SEC: Social Emotional Cognition Task
sLORETA: Standardized low-resolution brain electromagnetic tomography
TWOB: Two Back Task
