# Supplementary material for: Disrupted brain network dynamics and cognitive functions in methamphetamine use disorder: insights from EEG microstates
Source: BMC Psychiatry. 2020 Jun 24;20:334. doi: 10.1186/s12888-020-02743-5 (PMC7315471; doi:10.1186/s12888-020-02743-5)
Supplement: Supplementary file 1 — Additional file 1: Table S1. Brain regions that showed increased activation of microstate A at rest in patients with methamphetamine use disorder. Table S2. Brain regions that showed increased activation of microstate B at rest in patients with methamphetamine use disorder. Table S3. Brain regions that showed increased activation of microstate C at rest in patients with methamphetamine use disorder. Fig. S1. sLORETA differences in the cortical distribution of electrical activity sources of microstate class A between patients with methamphetamine use disorder and health controls. Significant differences are displayed (yellow = patients>controls). Fig. S2. sLORETA differences in the cortical distribution of electrical activity sources of microstate class B between patients with methamphetamine use disorder and health controls. Significant differences are displayed (yellow = patients>controls). Fig. S3. sLORETA differences in the cortical distribution of electrical activity sources of microstate class C between patients with methamphetamine use disorder and health controls. Significant differences are displayed (yellow = patients>controls). [file 12888_2020_2743_MOESM1_ESM.docx]

*BMC Psychiatry: Research Article*

Disrupted brain network dynamics and cognitive functions in methamphetamine use disorder: Insights from EEG microstates

Tianzhen Chen^1, *^, Hang Su^1, *^, Na Zhong^1^, Haoye Tan^1^, Xiaotong Li^1^, Yiran Meng^2^, Chunmei Duan^2^, Congbin Zhang^2^, Juwang Bao^3^, Ding Xu^4^, Weidong Song^4^, Jixue Zou^5^, Tao Liu^6^, Qingqing Zhan^6^, Haifeng Jiang^1, 7, #^, Min Zhao^1, 7, 8, 9^

^1^Shanghai Mental Health Center, Shanghai Jiao Tong University School of Medicine, Shanghai, China.

^2^Yunnan Institute on Drug Dependence, Yunnan, China.

^3^Institute of higher education, Beijing university of technology, Beijing, China.

^4^Shanghai Bureau of Drug Rehabilitation Administration, Shanghai, China.

^5^Department of Health, Yunnan Bureau of Drug Rehabilitation Administration, Yunnan, China.

^6^Yunnan Third Compulsory Drug Dependence Rehablitation Center Hospital, Yunnan, China.

^7^Shanghai Key Laboratory of Psychotic Disorders, Shanghai, China.

^8^Institute of Psychological and Behavioral Science, Shanghai Jiao Tong University, Shanghai, China.

^9^CAS Center for Excellence in Brain Science and Intelligence Technology (CEBSIT), Chinese Academy of Sciences, Shanghai, China.

*These authors contributed equally.

#Corresponding author:

Haifeng Jiang, M.D., Ph.D.,

Shanghai Mental Health Center, Shanghai Jiao Tong University School of Medicine, 600 Wan Ping Nan Road, Shanghai 200030, China. E-mail: dragonjhf@hotmail.com

**Supplementary Material**

| Table S1 Brain regions that showed increased activation of microstate A at rest in patients with methamphetamine use disorder. | | | |
| --- | --- | --- | --- |
| Brain Region | Brodmann area | Number of voxel | |
|  |  | left | right |
| Inferior Parietal Lobule | 7, 39, 40 | 56 | 110 |
| Middle Frontal Gyrus | 6, 8, 9, 10, 46 | 2 | 31 |
| Middle Temporal Gyrus | 39 | - | 1 |
| Superior Temporal Gyrus | 13, 22, 39, 42 | 5 | 17 |
| Superior Parietal Lobule | 5, 7 | - | 3 |
| Precentral Gyrus | 4, 6 | 30 | 50 |
| Supramarginal Gyrus | 39, 40 | 5 | 24 |
| Insula | 13 | 1 | 4 |
| Left and Right represent Left and Right hemisphere, respectively. | | | |

| Table S2 Brain regions that showed increased activation of microstate B at rest in patients with methamphetamine use disorder. | | | |
| --- | --- | --- | --- |
| Brain Region | Brodmann area | Number of voxel | |
|  |  | left | right |
| Middle Occipital Gyrus | 18, 19 | 26 | - |
| Inferior Parietal Lobule | 7, 39, 40 | 107 | 42 |
| Inferior Frontal Gyrus | 9, 11, 47 | 4 | 22 |
| Cuneus | 7, 18, 19 | 40 | 41 |
| Precuneus | 7, 19, 23, 31, 39 | 19 | 23 |
| Subcallosal Gyrus | 13 | - | 2 |
| Sub-Gyral | 6 | 1 | - |
| Superior Occipital Gyrus | 19 | 8 | 2 |
| Angular Gyrus | 39 | 15 | 11 |
| Supramarginal Gyrus | 39, 40 | 28 | 4 |
| Left and Right represent Left and Right hemisphere, respectively. | | | |

| Table S3 Brain regions that showed increased activation of microstate C at rest in patients with methamphetamine use disorder. | | | |
| --- | --- | --- | --- |
| Brain Region | Brodmann area | Number of voxel | |
|  |  | left | right |
| Inferior Frontal Gyrus | 9, 11, 44, 45, 46, 47 | 61 | 72 |
| Inferior Temporal Gyrus | 19, 20, 21, 37 | 49 | 64 |
| Cingulate Gyrus | 23, 24, 31, 32 | 24 | 22 |
| Insula | 13 | 15 | 16 |
| Posterior Cingulate | 18, 23, 29, 30, 31 | 39 | 32 |
| Inferior Parietal Lobule | 40 | 73 | 126 |
| Lingual Gyrus | 17, 18, 19 | 66 | 73 |
| Orbital Gyrus | 47 | 4 | 5 |
| Parahippocampal Gyrus | 19, 27, 28, 30, 34, 35, 36, 37 | 89 | 49 |
| Supramarginal Gyrus | 39, 40 | 8 | 27 |
| Rectal Gyrus | 11 | 5 | - |
| Precentral Gyrus | 4, 6 | 26 | 51 |
| Left and Right represent Left and Right hemisphere, respectively. | | | |


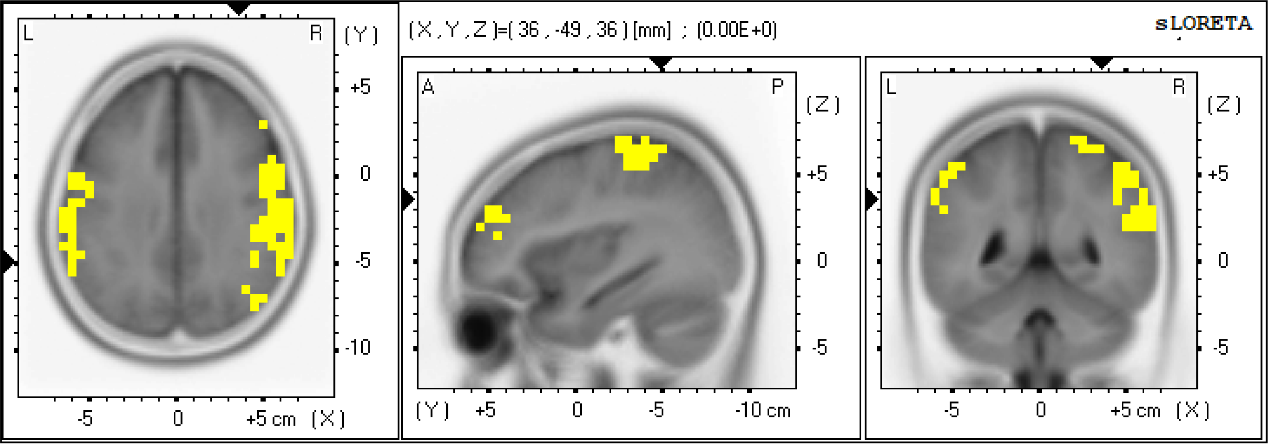


Fig. S1 sLORETA differences in the cortical distribution of electrical activity sources of microstate class A between patients with methamphetamine use disorder and health controls. Significant differences are displayed (yellow=patients>controls).


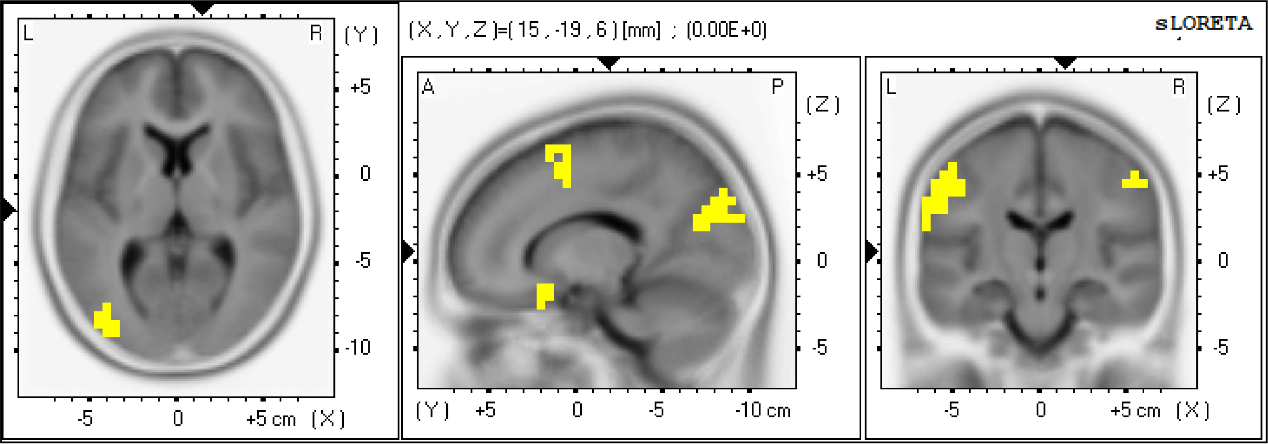


Fig. S2 sLORETA differences in the cortical distribution of electrical activity sources of microstate class B between patients with methamphetamine use disorder and health controls. Significant differences are displayed (yellow=patients>controls).


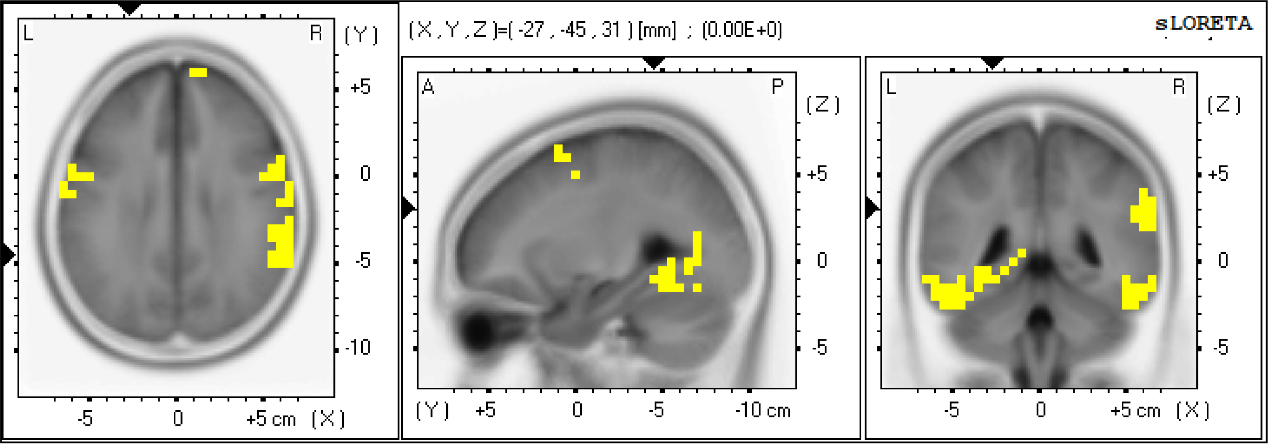


Fig. S3 sLORETA differences in the cortical distribution of electrical activity sources of microstate class C between patients with methamphetamine use disorder and health controls. Significant differences are displayed (yellow=patients>controls).
